# *Sclerotinia sclerotiorum* (Lib.) de Bary: Insights into the Pathogenomic Features of a Global Pathogen

**DOI:** 10.3390/cells12071063

**Published:** 2023-03-31

**Authors:** Md. Motaher Hossain, Farjana Sultana, Weiqiang Li, Lam-Son Phan Tran, Mohammad Golam Mostofa

**Affiliations:** 1 Department of Plant Pathology, Bangabandhu Sheikh Mujibur Rahman Agricultural University, Gazipur 1706, Bangladesh; 2 College of Agricultural Sciences, International University of Business Agriculture and Technology, Dhaka 1230, Bangladesh; 3 Jilin Da’an Agro-Ecosystem National Observation Research Station, Changchun Jingyuetan Remote Sensing Experiment Station, State Key Laboratory of Black Soils Conservation and Utilization, Northeast Institute of Geography and Agroecology, Chinese Academy of Sciences, Changchun 130102, China; 4 Institute of Genomics for Crop Abiotic Stress Tolerance, Department of Plant and Soil Science, Texas Tech University, Lubbock, TX 79409, USA; 5 MSU-DOE Plant Research Laboratory, Michigan State University, East Lansing, MI 48824, USA

**Keywords:** cAMP, cell wall degrading enzymes, effectors, life cycle, molecular diagnosis, oxalic acid, pathogenicity, whole genome

## Abstract

*Sclerotinia sclerotiorum* (Lib.) de Bary is a broad host-range fungus that infects an inclusive array of plant species and afflicts significant yield losses globally. Despite being a notorious pathogen, it has an uncomplicated life cycle consisting of either basal infection from myceliogenically germinated sclerotia or aerial infection from ascospores of carpogenically germinated sclerotia. This fungus is unique among necrotrophic pathogens in that it inevitably colonizes aging tissues to initiate an infection, where a saprophytic stage follows the pathogenic phase. The release of cell wall-degrading enzymes, oxalic acid, and effector proteins are considered critical virulence factors necessary for the effective pathogenesis of *S. sclerotiorum*. Nevertheless, the molecular basis of *S. sclerotiorum* pathogenesis is still imprecise and remains a topic of continuing research. Previous comprehensive sequencing of the *S. sclerotiorum* genome has revealed new insights into its genome organization and provided a deeper comprehension of the sophisticated processes involved in its growth, development, and virulence. This review focuses on the genetic and genomic aspects of fungal biology and molecular pathogenicity to summarize current knowledge of the processes utilized by *S. sclerotiorum* to parasitize its hosts. Understanding the molecular mechanisms regulating the infection process of *S. sclerotiorum* will contribute to devising strategies for preventing infections caused by this destructive pathogen.

## 1. Introduction

*Sclerotinia sclerotiorum* (Lib.) de Bary is among the world’s most virulent and widespread plant-killing organisms. This fungus is widely distributed throughout temperate, tropic, and arid regions [1]. The disease caused by this fungal pathogen is named by various terms, including white mold, watery soft rot, cottony rot, cottony soft rot, drop, stem rot, bloom blight, crown rot, and *Sclerotinia* rot [2]. Among these terms, white mold is most frequently used to refer to the disease caused by *S. sclerotiorum*. *S. sclerotiorum* possesses profound parasitic abilities and can colonize nearly all plant tissues with its mycelia. Critically, this fungus does not have a specificity for any particular host and infects over a few hundred monocotyledonous and dicotyledonous plant species, including some economically important crops, flowers, and weeds [2,3,4,5,6,7,8,9]. While *S. sclerotiorum* is primarily a plant parasite, a recent study has found that it can also grow endophytically in monocots, including rice (*Oryza sativa*), wheat (*Triticum aestivum*), maize (*Zea mays*), barley (*Hordeum vulgare*), and oat (*Avena sativa*) [10].

As a plant pathogen, *S. sclerotiorum* is highly destructive, and its infection frequently results in significant crop damage and yield loss. Infected vegetable crops become entirely unvendible, and infected grain and oilseed crops suffer from decreased seed weight, number, and/or quality, eventuating substantial economic losses [3,11]. Losses caused by this pathogen can vary significantly based on geographic locations and crops. In favorable environments, yield losses often surpass 20–35%, although cases of over 50% and as high as 80–100% have been documented in various places, particularly in temperate climates [12]. The lack of effective host resistance, vast host range, and general difficulties in dealing with this disease are the primary reason for *S. sclerotiorum*-caused plant damage.

*S. sclerotiorum*, being a belligerent necrotroph, can effectively cause cell death in plant tissues. The fungus produces numerous lytic enzymes, including endo- and exo-pectinases, cellulases, hemicellulases, and proteases, which aid in colonization and cause the breakdown of host cell walls [13]. Alongside cell wall-degrading enzymes (CWDE), oxalic acid (OA) has a central role in pathogenesis [14]. Pathogen proteins, or effectors, are secreted by the fungus and are also considered essential factors for its pathogenesis. After infection, a series of carefully regulated events results in programmed cell death of the hosts and, thus, disease resistance. Significant research has concentrated on the mechanisms and processes used by *S. sclerotiorum* to infect host cells. The annotated *S. sclerotiorum* genome is a significant treasure, which can substantially be used to elucidate the complicated mechanisms of pathogenesis caused by this fungus. This review aims to focus on recent advances in understanding the genetic and genomic elements that contribute to *S. sclerotiorum* pathogenicity in host plants.

## 2. Unique Signs and Symptoms That Constitute Clinical Features of *S. sclerotiorum*

**Figure 1:**
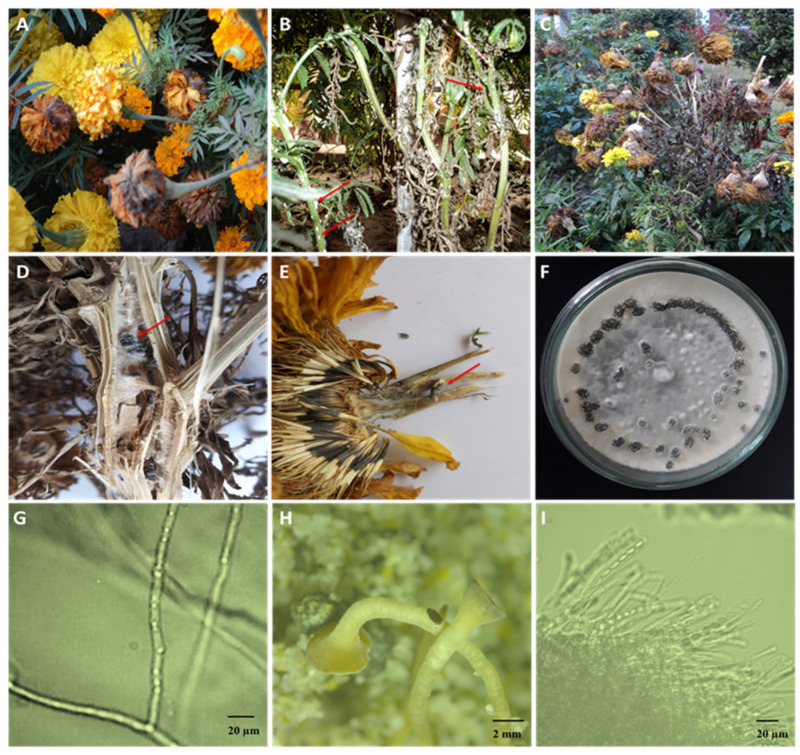
Infected marigold plants and the isolated fungus. (**A**) Infected marigold plants showing white mycelium growth (indicated by arrows). (**B**) Infected flower buds showing white rot symptoms. (**C**) Severely infected garden showing infected, wilted, and dried plants. (**D**) Infected stem showing sclerotial development in the internal pith tissues (indicated by arrows). (**E**) Infected marigold flower with external and internal brown discoloration with embedded black sclerotia (indicated by arrows). (**F**) Pure culture of the infected fungus showing fluffy white mycelium and sclerotial ring. (**G**) Micrograph of the mycelium of *Sclerotinia sclerotiorum* isolated from marigold plants. (**H**) Induced formation of mature apothecia. (**I**) Rows of asci containing ascospores.

Correct disease identification at the onset is a crucial step in pathogen control and disease management. Traditionally, agricultural extension workers and other experts identify disease by observing signs and symptoms produced by a pathogen. Since *S. sclerotiorum* has a broad host range, no symptoms are exclusive to all infected plants. White mold is easily identifiable on any host by white cottony *S. sclerotiorum* mycelium on affected aerial tissues [15]. Stems, leaves, petioles, and reproductive organs may initially show water-soaked lesions with a distinct margin, followed by wilting, bleaching, and shredding. The cottony hyphae congregate into pea-sized clumps of mycelium, which gradually mature into hard black sclerotia, typically on the infected tissue’s surface and inside soft host tissues or cavities [15]. In sunflowers, the first aboveground symptom may be sudden wilting before or during flowering, resulting from root rot or basal stalk rot near the soil line [16]. Infections may occur on stems at any time of growth, leading to stem rot. The fungus also attacks sunflower head, causing the inner head to rot, disintegrate, and shred, leaving behind large sclerotia [16]. The symptoms of canola include the appearance of bleached, greyish lesions on the main stem, branches, or pods; the presence of hard, melanized, black sclerotia within the cortex of infected stems; and the premature flowering and wilting of plant tissues in the terminal parts of infected stems [11]. Infected stems are often prone to lodging during flowering and seed filling. In marigolds (*Tagetes erecta*), the first symptoms are typically visible during flowering. The symptoms appear on the flower petals and then spread to the entire flower and the lower section of the flower (Figure 1A). Infection may migrate from the infected flowers to stems or leaves in close proximity to the infected flowers. Leaves often develop water-soaked lesions that rapidly grow and become gray-green before turning dark brown and dying. Some infected plants may acquire whitish fungal growth on their stems and exhibit water-soaked lesions (Figure 1B). Infected stems may die prematurely, turn tan, and eventually bleach. The severe infection makes the plant weak and can cause it to wilt, fall over, and eventually die (Figure 1C). During humid conditions, infected plant parts often show signs of *S. sclerotiorum*, such as whitish, fluffy mycelium and big, irregularly shaped sclerotia on the host surface or embedded in the infected tissues (Figure 1D,E). Sclerotia are variable in shape but are generally globose to cylindrical, ranging from 2.0 to 20.0 mm [2,5,17]. On PDA, colonies rapidly expand and have white, cottony, aerial mycelia with a salmon-buff hue on the back. At the expanding edges of the colonies, sclerotia form radiating lines and concentric rings (Figure 1F). The hyphae are hyaline, branched, and multinucleate (Figure 1G). A sclerotium produces one or multiple apothecia of a tan to amber hue (Figure 1H). Apothecian asci are cylinder-shaped and have eight spores (Figure 1E). The ascospores are uniseriate, unicellular, hyaline, and elliptical (Figure 1F), whereas the paraphyses are abundant.

## 3. A Naive Lifecycle of a Deadly Pathogen

**Figure 2:**
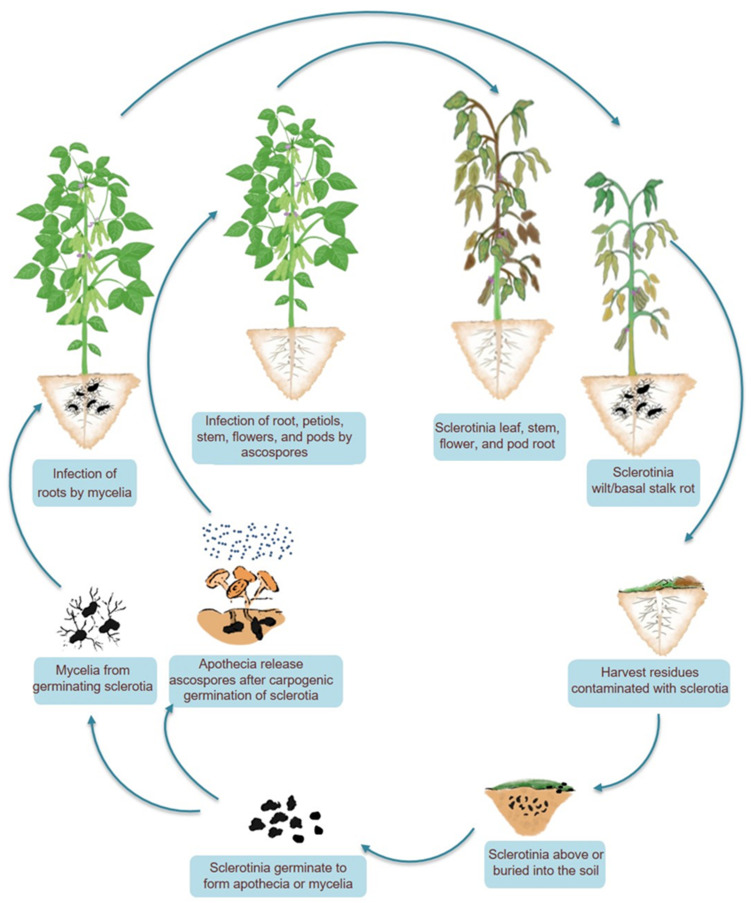
Life cycle and signs of infection of *Sclerotinia sclerotiorum* in soybean. The fungus has a monocyclic life cycle and does not produce secondary inoculum. It forms a survival structure known as a sclerotium on or inside host tissues, allowing it to thrive in soil. Sclerotia germinate and give rise to apothecia or mycelia. Mycelia infect roots or stem bases, whereas wind-transported ascospores, after being liberated from the asci, infect aerial parts, such as stems, foliage, buds, flowers, and fruits. At the end of the growing season, *S. sclerotiorum* produces sclerotia, which persist on the ground surface or in the soil, on either living or dead plant tissues, until the following season.

Because there is only one cycle of inoculum production during the course of infection, *S. sclerotiorum* is a simple monocyclic pathogen (Figure 2). The *S. sclerotiorum* lifecycle is primarily anchored by the formation of sclerotium. Sclerotia remain dormant in the soil for most of the *S. sclerotiorum* lifecycle and serve as the principal surviving structure. Sclerotium germinates directly (myceliogenic) or indirectly (carpogenic) and signals the inception of a new cycle of disease. Myceliogenic germination (by mycelium), possibly triggered by root exudates, is the primary mode of infection and is important from an epidemiological standpoint [18]. Mycelia from soilborne sclerotia may directly initiate infection on roots and basal stem. Basal stem rot and wilt, and in extreme cases, plant death, are the outcomes of the colonization of lateral roots, tap roots, and stem bases [18]. Ascospores released following carpogenic germination of sclerotia may also induce infection in plants. Despite being vulnerable to predation and deterioration from microorganisms, each sclerotium has the ability to produce carpogenic germination and several stipitate apothecia over multiple years. Each apothecium generates clusters of ascospores that are forcibly expelled into the air and disseminated within and between fields of vulnerable crops. The airborne ascospores are primarily associated with the infection of aerial plant parts [19].

In flower plants, ascospores that land in the sugary secretions of the extrafloral nectaries adjacent to the leaf margins germinate and colonize the leaf, leafstalk, and, eventually, the stem, causing stem rot [18]. During blossoming, the deposition of ascospores among the florets can result in the colonization of the capitulum, which causes bud rot (Figure 1). When conditions are optimum, the infection extends from the site of initial infection to the rest of the plant and then to uninfected plants. The potential of ascospores for airborne distribution and infection presents a unique limitation for the fungus since ascospores cannot infect healthy tissues. Ascospores are able to germinate, even invade host tissues, and cause compatible infections only when dead or senescent tissues are colonized saprotrophically before infecting healthy tissues [20]. In the field, this is typically done by colonizing leaves and flower petals that have senesced, damaged, or abscised but are still in physical contact with uninfected healthy host tissue (Figure 2). Mycelial growth from sclerotia on the soil surface can also initiate infection. The germinated hyphae colonize dead organic matter and then infect neighboring alive plants [21]. The life cycle is completed by forming sclerotia outside or inside hollow stems, flowers, buds, and fruits that have been colonized by the fungus (Figure 2). Due to the extended soil survival of sclerotia, even in adverse conditions, controlling white mold is difficult [22]. The sclerotium of *S. sclerotiorum* consists of three distinct layers, making this multihyphal structure hardy and enduring. The thick outer black rind contains melanin, a substance considered significant in the protection against unfavorable circumstances and microbial decomposition, and in some fungi, it has functions in virulence [23]. However, for *S. sclerotiorum*, no connection between melanin and virulence has so far been known.

The medulla, the innermost section, is embedded in a fibrillar matrix and is mainly made of proteins and glucans. Three phases of sclerotial formation have been identified: the first phase is initiation or commencement (hyphae aggregate to generate a white sclerotial mass or initials); the second phase is development (sclerotial initials increase in size); and the third phase is maturation (surface delimits, melanin deposits in peripheral rind cells, and internal cells consolidate) [2,5,17]. Several factors, including many dietary elements, environmental changes, secondary messengers, and primary metabolism, affect sclerotial formation and development. Sclerotia are typically formed when mycelial development confronts a nutrient-limited environment [17]. Glucose, yeast extract, peptone, ascorbic acid, and casein are the most suitable sources of nutrients for sclerotial formation in *S. sclerotiorum* [2,5,17]. Moreover, sclerotial development is favored in media enriched with calcium, magnesium, potassium, and sodium [2]. Temperature alone or in conjunction with other variables may affect sclerotial growth and survival. A temperature of 20 °C promotes maximum sclerotial development, while sclerotial production is significantly constrained at temperatures above 25 °C and, to a lesser degree, below 20 °C [2]. Consequently, low temperatures have been linked to the development of *Sclerotinia* infections in various plants [24]. pH has been demonstrated to affect sclerotial growth significantly. Sclerotial production is hindered at a neutral or alkaline pH [14], while it is favored at an acidic pH [2,5]. In contrast, fungus accumulation of OA lowers the pH and promotes sclerotial formation [14]. In line with this notion, *S. sclerotiorum* mutants incapable of producing OA do not form sclerotia in vitro and infect planta [25]. However, reducing the ambient pH in these mutants does not restore their ability to form sclerotia [14], indicating the involvement of more factors than pH alone. A number of genetic regulators are known to control the formation and development of sclerotia, which will be discussed in the later part of the current review.

## 4. Two-Phase Infection Model: The Transition from Biotrophy to Necrotrophy

**Figure 3:**
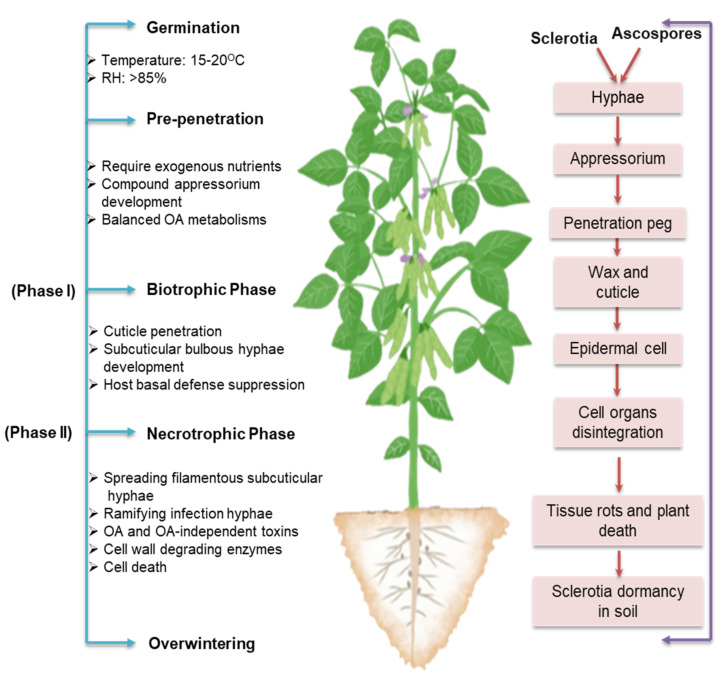
A two–phase infection model of *Sclerotinia sclerotiorum* presented schematically, with significant cellular events noted for each infection phase. During phase one, the pathogen employs well-planned techniques to overcome host basal defense mechanisms and establish fundamental compatibility. This is accomplished by forming infection structures, such as compound appressoria, bulbous subcuticular hyphae, and primary invasive hyphae. In phase two, the pathogen uses toxins, particularly oxalic acid (OA) and hydrolytic enzymes, to disrupt and degrade host tissues, resulting in rapid tissue maceration and death.

Although *S. sclerotiorum* has been widely accepted as a necrotrophic pathogen, recent evidence demonstrates that it is, in fact, a hemibiotroph [26]. In the early phases of infection, once *S. sclerotiorum* has penetrated the cuticle, the spread does not cause host cell necrosis. There is a brief period for biotrophic development in the apoplastic gaps prior to the necrotization of host cells. In this two-phase infection model, as described by Liang and Rollins [26], *S. sclerotiorum* evades, thwarts, and suppresses host basal defense responses before killing and destroying host cells (Figure 3).

The development of modified hyphae into appressoria is crucial for the infection of healthy tissues. During field infection, these hyphae are produced either directly from sclerotia or indirectly from ascospores in the presence of external nutrients, primarily from flowers or other aging tissues [27]. Depending on the host surface, simple or complex appressoria can be formed. Simple appressoria originate from dichotomous terminal branching of hyphae growing on the host surface. These appressoria are made up of a pad of broad, multiseptate, and short hyphae that are fixed perpendicularly to the surface of the host by mucilage [21]. A compound appressorium or infection cushion forms from a hyphal tip during recurring processes, such as swelling, growth retardation, and successive bifurcation [26]. The development of infection cushions is thought to improve the adherence of the pathogen to the host surface [28]. Previous research has suggested that infection cushion aids in penetrating the cuticle barrier of the host epidermis through mechanical pressure and/or enzymatic disintegration of the host surface during compatible interaction [29]. While in incompatible interaction, the most important component of the defense mechanisms by which the host avoids penetration and colonization by *S. sclerotiorum* is the active suppression of infection cushions [30], indicating the core role of infection cushions in the infection process of the fungus. Hyphae that form infection cushions frequently become flattened and larger in diameter, from which thin penetration pegs develop. Penetration of a host tissue only occurs beneath infection cushions and/or compound appressorium [28,30]. Hyphal tips are compactly aligned within a compound appressorium [28]. Separate hyphal tips initiate separate penetrations. The localized grouping of multiple hyphal points within a continuous plane, as opposed to a single-celled appressorium, likely increases the local amassing of toxins, hydrolytic enzymes, and defense-inhibitory substances [26]. With mechanical force and enzymatic digestion, penetration pegs derived from appressorium penetrate the cuticle layer, but not the underlying epidermal cells of the host [28,31]. Bulbous and multilobed subcuticular vesicles arising from these penetration pegs are responsible for the production of subcuticular infection hyphae, which grow in a horizontal direction beneath the cuticle to form the leading front of the colonization process [26].

Subcuticular hyphae of *S. sclerotiorum* spread through several cell layers before epidermal cells are killed [31,32], implying that *S. sclerotiorum* infection includes a transitory biotrophic phase, which begins around 12–24 h after infection [33]. During this phase, compatibility is established between the host and the pathogen primarily through suppressing or subverting host physical and chemical defense barriers. Effector genes in the pathogen *S. sclerotiorum* are upregulated, which may inhibit host–pathogen recognition and host defense mechanisms [34]. This initial phase is crucial for the “necrotrophic” success of the pathogen. After the initial growth of subcuticular hyphae, smaller hyphae ramify from the subapical ends and grow between and inside epidermal and mesophyll cells [26]. With the successful colonization by ramifying hyphae, *S. sclerotiorum* enters the necrotrophic phase [34], during which it produces significant amounts of reactive oxygen species (ROS), toxins, and CWDEs, which result in the dissolution of the subcuticular epidermal walls, rapid cell death, and development of necrotic symptoms [33,35,36].

## 5. Privileges of Massive Arsenal to Subsist and Succeed against Host Defense

**Figure 4:**
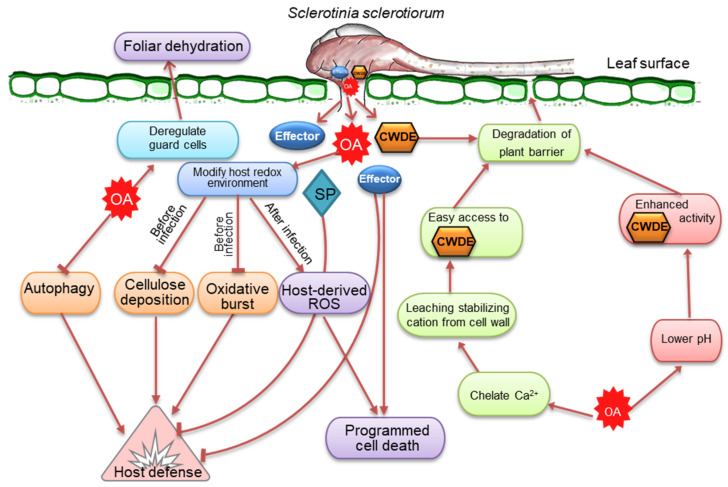
The frontline weapons or virulence factors of *Sclerotinia sclerotiorum* important for developing and regulating the infection process in plants. *S. sclerotiorum* releases oxalic acid (OA), which modulates reactive oxygen species (ROS) and the activity of cell wall-degrading enzymes (CWDEs) while dampening host defense responses and suppressing autophagy. At the early stage, OA suppresses plant defensive responses and modifies the host redox state by inhibiting callose deposition and host oxidative burst and establishing a reducing environment. At a later stage, OA-induced ROS causes apoptotic-like programmed cell death and disease. Because of its affinity for Ca^2+^, OA may degrade plant barriers by draining the stabilization of cations from the plant cell wall. OA accumulation also lowers the pH and activates CWDEs, which are necessary to destroy infection surfaces. Effectors and other secreted proteins (SP) have a similar role in cell death and host defensive responses.

*S. sclerotiorum* possesses an immense arsenal of disease weaponry to subsist and succeed under widespread environmental conditions. The massive pathogenic arsenal of *S. sclerotiorum*, including oxalic acid (OA), CWDEs, and small secretory proteins (effectors), has long been associated with virulence [26]. Recent research reveals that a subtle interplay between these virulence factors serves various regulatory functions in host cells, allowing *S. sclerotiorum* to colonize the host, evade or inhibit the host defense system, and cause disease (Figure 4). Understanding and studying these mechanisms is crucial for detecting pathways of genetic interventions that could result in improved control of this disease.

### 5.1. Oxalic Acid, a Versatile Toxin and Broad-Spectrum Pathogenicity Factor

Of the various virulence factors, extensive research has been conducted on the relationship between white mold disease and the production of OA by *S. sclerotiorum* [37,38]. This fungus is well-known for its ability to acidify the surroundings via OA secretion. The OA-deficient mutants of the fungus have been shown to be poorly pathogenic [39], suggesting that the pathogenesis of *S. sclerotiorum* requires accumulating high quantities of OA. While OA-minus mutants can still infect and colonize hosts to variable degrees, it is appropriate to view OA more as a colonization factor rather than a crucial compatibility element [31]. OA is not directly toxic to plant cells but plays a more sophisticated role as a signaling molecule that seizes control of host cells and creates an environment favorable for *S. sclerotiorum* growth. OA alters a number of physiological processes in host plants. OA promotes cell wall degradation by increasing the activity of polygalacturonases, inhibiting plant-protection enzymes, subduing oxidative burst, deregulating stomatal guard cell closure, mediating pH signaling, inducing apoptosis-like cell death, and altering cellular redox status in plants (Figure 4) [14,35,39,40,41,42,43]. The production of OA seems to suit the overall strategy of *S. sclerotiorum* to acidify its ambient environment to facilitate infection in several ways. Many of the fungal enzymes (such as polygalacturonase) secreted during the invasion of plant tissues have maximal activities at a low pH [40]. It is postulated that OA could increase *Sclerotinia* virulence by adjusting the apoplastic pH to a level conducive to the enzymatic breakdown of plant cell walls. OA can also weaken plant cell walls via acidity, thereby facilitating invasion [40]. Interestingly, no ROS production is detected at acidic pH [43]. It is evident that *S. sclerotinia* uses OA to elicit apoptosis-like programmed cell death, and the induction of this process is commenced by a reducing environment generated by the fungus in the host cell [35]. As a direct consequence of this redox manipulation, the fungus subverts host defense responses, inhibits oxidative burst, and prepares the infection court for disease establishment [35]. These findings imply that the effect of OA on the reduction of pH in the ambient environment is significantly greater than that of OA alone as an essential component of the infection process caused by *S. sclerotiorum*. Intriguingly, the regulation of host defense by OA appears to be biphasic. In the early phases of infection, OA suppresses host defenses by culminating ROS buildup and callose deposition in the host [35]. OA sequesters Ca^2+^ liberated during cell wall collapse, thereby protecting the development of *S. sclerotiorum* hyphae from hazardous Ca^2+^ concentrations [44]. In the later stages of infection, OA quashes antioxidant enzyme activities and stimulates ROS generation, concurrently damaging the membranes of host cells [45]. These findings suggest that OA is a cross-functional toxin crucial to the widespread pathogenic success of *S. sclerotiorum*.

The dicarboxylic acid OA can be produced through the oxidation of glyoxylate or glycolaldehyde or the hydrolysis of oxaloacetate [46]. However, current research indicates that OA biosynthesis entirely depends on the oxaloacetate acetylhydrolase (OAH) activity [26]. OAH mediates the C-C cleavage of oxaloacetate during the OA biosynthesis process [31]. Depending on the growth substrate, either the tricarboxylic acid (TCA) cycle or the glyoxylate cycle may produce oxaloacetate precursor [47]. Experimental evidence from *S. sclerotiorum* and *B. cinerea* gene-deletion mutants supports the hypothesis that the expressed enzyme activity is responsible for catalyzing the last step in the OA biosynthesis process [48]. Three types of OA-degrading enzymes are found throughout evolutionary lineages: OxDC (EC 4.1.1.2), OxOx (EC 1.2.3.4), and oxalylCoA decarboxylase (OXC, EC 4.1.1.8). *S. sclerotiorum* exhibits oxalate decarboxylase (OxDCs) activity but not OxOx [49], while only bacterial species have been identified to possess OXC activity. OxDC catalyzes the production of formate and CO_2_ from OA, and its activity is widespread among fungi and bacteria [50].

### 5.2. Arsenal of Cell Wall-Degrading Enzymes Reflects Broad Host Preference

An essential component of the lifecycle of *S. sclerotiorum* is its capacity to break down a wide range of complex plant polysaccharides by CWDEs (Figure 4). Cellulose, hemicellulose, and pectin form a complex network of polysaccharides that constitute plant cell walls [51]. This network is the target of CAZymes, a group of proteins and enzymes that break down plant cell wall polysaccharides into simple monomers [52]. These monomers serve as carbon sources and allow access to internal plant tissues. At least 118 CAZymes are explicitly associated with plant cell wall degradation by *S. sclerotiorum*; however, many other CAZymes presumably contribute to this activity [53]. Several endo- and exo-polygalacturonases (PGs) are known to break down unesterified pectate polymers, middle lamella structural polysaccharides, and primary plant cell wall components. Endo-PGs catalyze the hydrolysis of homogalacturonan, whereas exo-PGs fragment the substrate and release potential nutrients by removing monomeric or dimeric glycosyl groups from pectic cell wall polysaccharides [54]. PGs are also recognized as potential virulence factors in several pathosystems [55,56,57]; however, convincing proof for their universal involvement in virulence is missing. The principal hemicellulose constituent of plant cell walls, xylan, is comprised of xylose and arabinose connected by β-(1–4)-linked xylopyranose. Endo β-1, 4-xylanase is the major enzyme that degrades xylan [58]. Studies have highlighted the critical functions of endo-β-1, 4-xylanase in the development and pathogenicity of *S. sclerotiorum* [59]. Other important groups of mannan- and cellulose-degrading enzymes are also highly upregulated [53]. These indicate that *S. sclerotiorum* utilizes a diverse array of enzymes that play significant roles in its infection strategy across many host species.

### 5.3. Secretory Effectors, Critical Components in the Necrotrophic Infection Strategy

Numerous small, secreted proteins of *S. sclerotiorum* have been reported as having effector-like features or effector-like activities, which are released either extracellularly or within the cytoplasm of plant cells to disarm host defense mechanisms and enhance fungal infection (Figure 4) [33,60]. Until now, a number of *S. sclerotiorum* effectors associated with virulence have been identified. During the infection of soybean seedlings, *S. sclerotiorum* produces a basic endopolygalacturonase (PG) isoform early in the process that elicits Ca^2+^-mediated signaling and programmed cell death [61]. A polygalacturonase (PG) enzyme, called *Sspg1d*, reacts with proteins that have a C2 domain in *Brassica napus* [62]. Necrosis and ethylene-inducing peptides encoded by two genes, *SsNep1* and *SsNep2*, induce necrosis in tobacco (*Nicotiana tabacum*) leaves during heterologous expression [63]. A protein with a signal peptide and 302 amino acid residues in the integrin alpha N-terminal domain superfamily, SSITL, inhibits defense reactions in *Arabidopsis thaliana* [64]. A putative-secreted protein disrupting pathogen virulence has also been reported [65]. A cutinase encoded by *SsCut* induces cell death in an array of plants, including rice (*Oryzae staiva*)*, G. max*, *maize* (*Zea mays*), *B. napus*, wheat (*Triticum aestivum*), and *A. thaliana* [66]. A small cysteine-rich protein containing a cyanoviron-N homology (CVNH) domain, SsSSVP1, is required for pathogen virulence [67]. This putative effector is believed to alter plant energy metabolism in order to promote *S. sclerotiorum* infection. A small virulence-related protein 1, without obvious functional domains, has been found explicitly in *S. sclerotiorum* and *B. cinerea* [68].

In addition, several other effectors, such as chorismate mutase (*SsCM1*) [69], compound appressorium formation-related protein 1 (*SsCaf1*) [70], BAX inhibitor-1 (*SsBI1*) [71], superoxide dismutase (*SsSodI*) [72], catalase (*SsCat1*) [73], a protein analogous to a *Magnaporthe grisea* protein elicitor (*SsPemG1*) [74], and ceratoplantanin effector (*SsCP1*) [75], have been documented in *S. sclerotiorum*. The vast majority of the putative proteinaceous effectors characterized in *S. sclerotiorum* cause cell death in host tissue. *S. sclerotiorum* genome analysis has recently identified a novel class of necrosis-inducing effector 2 (*SsNE2*) [76]. On the contrary, a few biotrophy-related candidate effectors have been spotted, such as salicylate hydroxylase and a lysin motif (LysM) domain protein [33]. These effector genes were found to be upregulated within the first 24 h after infection. In biotrophic and hemibiotrophic pathogens, recognizing the effectors often leads to a hypersensitive reaction (HR) to contain the pathogens (Jones and Dangl, 2006) [77]. Contrariwise, for necrotrophic infections, the elicitation of HR responses by effectors delivers substrates to stimulate host colonization [26,78]. Additionally, *S. sclerotiorum* exhibits a host-specific expression of candidate effectors, as Guyon et al. [79] showed that the expression patterns of 16 effector candidates varied significantly between the hosts *S. lycospersicum*, *Nicotiana benthamiana*, and susceptible and resistant accessions of *A. thaliana*. According to previous studies, the host-specific expression of putative effectors was more limited than anticipated. Only one of the effector candidate genes (sscle 08g064180) identified by Derbyshire et al. [80] and Amselem et al. [53] was expressed differently between *B. napus* and *Lupinus angustifolius*, where the expression was significantly higher in *B. napus* compared to *L. angustifolius*. This putative effector is anticipated to have a coiled-coil domain, although little is known about the role of such domain in mediating effectors’ functions.

## 6. An Array of Genes Regulating Growth, Development, and Virulence

Molecular underpinnings of the genes that regulate mycelial growth, reproduction, and virulence in *S. sclerotiorum* have been the subject of several recent studies [26,37]. Because many of these genes have already been reviewed, the current review primarily focuses on works that have not been discussed in previous reviews [26,37]. A large number of genes required for *S. sclerotiorum* growth, development, and virulence have already been identified using the genome sequence and subsequent mutant analysis. These include *Sspac1* (pH-responsive transcription factor) [25], *Sscna1* (catalytic subunit calcineurin-encoding gene) [81], *PP2B* (type 2B phosphatase) [81], *Ssnox1*, *Ssnox2* (NADPH oxidase) [82], *SsITL* (integrin-like protein) [64], *Sssod1* (Cu/Zn superoxide dismutase) [83], *SsMADS* (MADS-box transcription factor) [84], *Scat1* (type A catalase) [73], *Shk1* (histidine kinases) [85], *SsCVNH* (cysteine-rich protein with a carbohydrate-binding module) [86], *Ssoah1* (oxaloacetate acetylhydrolase) [31], *Ssodc1* and *Ssodc2* (oxalate decarboxylase) [87], *SsBi1* (BAX inhibitor-1) [71], *Ss-Fkh1* (forkhead transcription factor) [88], *Sssop1* (microbial opsin homolog gene) [68], *SsXyl1* (Endo-β-1, 4-xylanase) [59], *SsSSVP1* (small secreted virulence-related protein) [67], *rhs1* (rearrangement hotspot repeat-containing protein) [89], *THR1* (trihydroxynaphthalene reductase) [90], *SCD1* (scytalone dehydratase) [26], *Ssams2* (GATA-box domain) [91], *Sssfh1* (GATA transcription factor with an SNF5 domain) [92], *SsSte12* (Ste12 transcription factor [37], *Sssvf1* (survival factor 1 homologue) [93], *Ssrhs1* (Rhs repeat-containing protein) [94], and *SsAGM1* (N-acetylglucosamine-phosphate mutase) [95] (Table 1). In mutant or transformant strains of *S. sclerotiorum*, knocking out or silencing these genes causes severe deficiencies in radial growth, sclerotial development, appressorium formation, virulence, and/or stress tolerance. For instance, the *pacC* gene homolog, a *Sspac1* replacement mutant, exhibited abnormal sclerotial development and maturation, implying that *Sspac1* is essential for the proper sclerotial development of *S. sclerotiorum* [25].

Likewise, *Ssoah1* mutants displayed a significant radial growth defect on buffered neutral pH substrate and failed to generate sclerotium or compound appressorium [31]. The nox (NADPH oxidase)-encoding gene, *Ssnox1*, contributes toward fungal virulence and sclerotial formation [82]. The MADS-box transcription factor, *SsMADS*, is essential for the growth and pathogenicity of *S. sclerotiorum* [84]. The *S. sclerotiorum sop1*, a microbial opsin homolog gene, is significantly upregulated during sclerotial development and infection relative to the vegetative growth stage [68]. Moreover, *sop1* is required for *S. sclerotiorum* growth, sclerotial development, full virulence, and stress responses. Because microbial opsins are missing in animals and most higher plant genomes, *sop1* is a candidate fungicide target for *S. sclerotiorum* control. The *S. sclerotiorum* integrin-like gene, *SsITL,* was intensely suppressed in the presence of *SsDRV*, a mycovirus gene linked with reduced virulence [64].

Silencing of *SsITL* decreased virulence, hyphal polarity, and qualitative and quantitative production of sclerotia in *S. sclerotiorum.* Sclerotia that were formed exhibited carpogenic germination deficiencies. In addition, *SsITL* encodes an effector involved in inhibiting jasmonate/ethylene-mediated host resistance during the initial phases of infection [64]. The function of *SsAMS2* in the development and growth of *S. sclerotiorum* has been investigated [91]. AMS2 protein is predicted to contain the GATA-box domain. *S. sclerotiorum* needs the transcription factor *SsAMS2* for growth, appressorium production, pathogenicity, and chromosome segregation [91]. Another gene with a GATA transcription factor with an SNF5 domain, *SsSFH1*, has also been analyzed and is known to influence growth, ROS accumulation, and pathogenicity of *S. sclerotiorum* [92].

According to recent research, vegetative development and sclerotial production in *S. sclerotiorum* are intimately tied to cell wall integrity. *SsFkh1* contributes to cell wall integrity, and the deletion mutants are significantly deficient in hyphal development and sclerotial formation [96]. *SsAGM1* has been isolated from several fungi, identified as necessary for chitin production, and known to play a role in preserving the integrity of cell wall [97,98]. Silencing *SsAGM1* substantially decreased the hyphal and sclerotial development of *S. sclerotiorum* [95]. The silenced transformants did not produce sclerotia, irrespective of the duration of the culture. In fact, *SsAGM1* mediates UDP-GlcNAc (uridine diphosphate-N-acetylglucosamine) synthesis, which influences mycelial development, cell wall integrity, cell wall chitin content, sclerotial production, infection cushion formation, and pathogenicity of *S. sclerotiorum* [95]. An Rhs repeat-containing protein (*Ssrhs1*) is shown to be imperative for hyphal infection and sclerotial development [94]. Some of these genes are components of a regulatory mechanism that mediates pH-responsive gene expression, indicating that pH-regulated gene expression is an essential feature of *S. sclerotiorum* growth, development, and virulence [25,31].

## 7. cAMP as a Key Modulator

Secondary messenger such as cyclic adenosine monophosphate (cAMP) also influences the growth, development, and virulence of *S. sclerotiorum*. cAMP is involved in the shift from mycelia to sclerotial growth [99]. An increase in either endogenous or exogenous cAMP suppresses sclerotial formation and stimulates OA accumulation [100]. In a ∆*bcg3* mutant of *B. cinerea* deficient in the Gα3 subunit, a drop in cAMP levels induced a substantial amount of sclerotial production under conditions that did not normally induce sclerotial production. On the contrary, sclerotial production in the ∆*bcg3* mutant was reduced by adding cAMP to the growth medium in a manner comparable to that seen in the wild type, suggesting that cAMP has a detrimental effect on sclerotial development [101]. cAMP-dependent PKA signaling was investigated to identify its potential functions in the pathogenesis, growth, and development of *S. sclerotiorum* [102]. In the *sac1*-disrupted mutant, the intracellular cAMP level was lowered by obliterating the single-copy adenylate cyclase gene (*sac1*) from the *S. sclerotiorum* genome. The disrupted mutant showed abnormal hyphal branching and the formation of excessive microconidia (spermatia) and aberrant sclerotia. Even though sclerotia generally form close to the outside edge of culture plates, the *S. sclerotiorum sac1* mutant generated sclerotia without ever reaching the edge of the culture plates. This demonstrated a negative role for cAMP in regulating the initiation of sclerotial formation. cAMP is also necessary for the development of compound appressorium and the pathogenicity of *S. sclerotiorum* [103]. cAMP has been suggested as a possible intermediary in the cellular cascade required for the production of a pathogenesis-related fungal protease known as Acp1 in *S. sclerotiorum* [104].

In other fungi, cAMP has been shown to have the opposite effect on sclerotial production. Higher intracellular cAMP levels facilitate sclerotial production in *Sclerotium rolfsii* and *Rhizoctonia solani* [99,105,106]. The enhancement of sclerotial development in an *R. solani* wild-type isolate was achieved by supplementing its growth medium with exogenous cAMP [99]. Hence, cAMP may have a species-specific role in sclerotial development. When sclerotial formation is triggered, the initial events are hyphal branching and fusion. cAMP likely influences sclerotial initiation by altering hyphal elongation and the rates of hyphal branching, supporting the observation that *Neurospora crassa* grows more slowly and more radially when intracellular cAMP levels are low [107]. The fact that hyphal branching is impaired in the *sac1* mutant, which has low cAMP levels and abnormal sclerotial formation, shows the association between cAMP levels, branching, and appropriate sclerotial development.

## 8. MAPK as Central Signaling Cascade

The mitogen-activated protein kinase (MAPK) is a major signaling cascade engaged in sclerotial formation and various processes related to fungal infection [37]. In *S. sclerotiorum*, an ERK-type MAPK, *Smk1*, was proven essential for sclerotiogenesis [108]. pH- and cAMP-dependent signaling mediates the regulation of sclerotial formation by MAPK [108]. The levels of cAMP-dependent protein kinase A (PKA) rise during sclerotial development in wild-type strains but remain low in mutants defective in sclerotial development [109]. However, similar pathogenicity, cAMP-responsiveness, and sclerotial development to wild-type strains were observed in *pka1* knock-out mutants, indicating a PKA-independent mechanism or the participation of other PKA-encoding genes in these processes [102]. As specific PKA inhibitors do not affect sclerotial development or cAMP-dependent MAPK inhibition, cAMP-mediated sclerotial suppression is PKA-independent [100]. Class III histidine kinases (HKs) are known to transmit signals via the high-osmolarity glycerol MAPK (HOG MAPK). The *S. sclerotiorum Shk1* gene, encoding a putative class III HK, plays a role in multiple processes, including vegetative differentiation, sclerotial formation, adaptation to stresses and fungicides, and glycerol accumulation in *S. sclerotiorum* [83]. Ras, a small GTPase that acts as an upstream activator of the MAPK pathway, is required for normal sclerotial growth because the loss of Ras function prevents MAPK activation [110]. Intriguingly, the suppression of a member of the Ras protein family, Rap-1, restores sclerotial growth and MAPK activation. These findings imply that sclerotial development depends on Ras/MAPK pathways, which are adversely controlled by Rap-1 in a PKA-independent cAMP signaling pathway [48]. In a recent study, the catalytic SsPKA and the regulatory SsPKAR subunits of the cAMP-dependent PKA signaling pathway in *S. sclerotiorum* were further characterized in terms of their functional properties [111]. Disruption of *Sspka2* or *SspkaR* markedly reduced growth and virulence, together with defects in appressorium development.

Unexpectedly, both disruptions exhibit an increase in autophagy without nutrient deprivation, indicating that accurately synchronized PKA activity is necessary to control autophagy. These findings show that the cAMP-dependent PKA signaling pathway disrupts autophagy and is vital in *S. sclerotiorum* development and virulence [111]. Investigation has been carried out to examine the potential downstream transcription factor of the MAPK pathway. *SsSte12* has been reported to be a transcription factor that acts as a downstream component of the MAPK pathway and is essential for a number of MAPK-dependent cellular processes, including penetration-dependent pathogenicity, mycelial growth, sclerotial development, and appressorium formation [37]. In reaction to different extracellular stimuli, MAPK is enzymatically stimulated by a series of phosphorylation events. It can also control the expression of genes and induce the phosphorylation of transcription factors [112]. Current research has demonstrated that the activity of protein phosphatase types 2A and 2B is reliant on MAPK and is needed for the development of sclerotia [81,113].

## 9. *Sclerotinia* Genome as a Treasure Trove

The first complete sequence of *S. sclerotiorum* genome was elucidated by Amselem and associates in 2011 [53]. This genome project used the isolate ‘1980’ collected from bean culls. Shotgun sequencing with Sanger sequencing chemistry and optical mapping was used to assemble the genome sequence. Genome annotation and gene validation employed expressed sequence tags (ESTs). These ESTs were obtained from key life cycle stages, such as sclerotium initials, mycelia, apothecia during the expansion of disc development, and compound appressoria. The assembled genome was 38 Mb, which covered 96% of the optical map. The majority of the unexplored areas on the map are likely centromeric locations. The number of predicted genes is 14,522, and the number of genes with a high degree of confidence is 11,860. The average GC contents found in the *S. sclerotiorum* genome are 41.8% [53]. Moreover, the *S. sclerotiorum* genome has eight duplicated blocks containing between four and twelve pairs of syntenic genes. Of the eight blocks, four contain genes encoding proteins. Approximately 7% of the genome is occupied by repetitive elements, with no apparent hotspots along the chromosomes. This expansion contains various categories of transposable components. LTR elements are commonly observed, followed by TIR, LINE, unknown, and MITE elements [53].

Derbyshire et al. [80] reported a complete and precisely annotated genome of the same *S. sclerotiorum* isolate ‘1980′. The new *S. sclerotiorum* genome assembly has an additional 805,146 base pairs compared to the previous genome assembly by Amselem et al. [53]. The majority of the extra bases constructed (78.7%) are within regions that are projected to be repeated. In addition, 12.96% of the genome is constituted of transposable elements, which is greater than the 7.7–9.0% previously estimated [80]. Though only a moderate improvement is shown in the new genome assembly, significant progress has been made in annotating new genes. If 11,860 unambiguous genes are considered in the previous genome version, there are yet 730 fewer in the new gene annotation set with 1130 predictions. Only 10,528 of the sequences with version one show reciprocal best BLAST matches, implying that an additional 602 sequences are either absent from the previous genome or have been altered significantly enough to prevent BLAST analysis from retrieving their corresponding loci as hits. The merging of previously separate loci is primarily responsible for the drop in filtered gene predictions. Recently, an upgraded genome assembly of *S. sclerotiorum* strain WH6 has been completed using the PacBio SMRT cell platform [114]. The assembled genome contains 10,512 predicted genes, with 685 secreted proteins and 65 candidate effectors, and is 38.96 Mbp in size with a contig N50 length of 1.90 Mbp. The *S. sclerotiorum* genome sequence from China had never been reported before the study of [114].

When comparing the related fungal genomes, *S. sclerotiorum* possesses a genome comparable in size and predicted gene number to *B. cinerea*, *Scleromitrula shiraiana*, and *Magnaporthe oryzae*, except for the biotrophic fungus *Blumeria graminis* f. sp. *hordei* (Table 2). The GC content in the *S. sclerotiorum* (41.55%) sequences is close to that of *B. cinerea* (42.0%) and *S. shiraiana* (38.9%). However, the percentage of repeated sequences in the *S. sclerotiorum* genome is greater than that of *S. shiraiana* (4.45%) and *B. cinerea* (4.4%). Similarly, the *S. sclerotiorum* genome (7%) contains more transposable elements than the genomes of *S. shiraiana* (0.07%) and *B. cinerea* (0.9%). The majority of the 11,860 predicted protein-coding genes are shared by *S. sclerotiorum* and *B. cinerea*., with an average similarity between these species of 84% for 8609 proteins [53]. Additionally, *S. sclerotiorum* shares 157,162 genes and 2605 ortholog families with 15 other fungi, including necrotrophs, biotrophs, and saprobes [115]. In the phylogenetic tree created with STRIDE (Species Tree Root Inference from gene Duplication Events) and STAG (Species Tree inference from All Genes) methods, *S. sclerotiorum* was grouped with the Leotiomycetes; *B. cinerea*, *S. shiraiana*, and *B. graminis* f. sp. *hordei*. Exclusively, *S. sclerotiorum, S. shiraiana*, and *B. cinerea* share 7402 ortholog families [115]. Although *S. sclerotiorum* is evolutionally nearer to *Scleromitrula shiraiana* and *B. cinerea*, the genome coverage remains at 72.3% [53] and 21.4% [115], respectively. Based on these results, it is plausible that *S. sclerotiorum* and *B. cinerea* diverged from their predecessors later in evolutionary time than *S. shiraiana* did.

A genome-wide search identified genes encoding key enzymes, including NRPS (non-ribosomal peptide synthetase), PKS (polyketide synthase), HYBRID (hybrid NRPS–PKS enzyme), DMATS (dimethylallyl tryptophan synthase) and TS (terpene synthase). Thirty-three key biosynthetic genes were found to encode these five enzymes in *S. sclerotiorum*, which were similar in number to those in *S. shiraiana*, but less than those in *B. cinerea* [115]. By analyzing the genes that are unique to *B. cinerea* and *S. sclerotiorum* and understanding the lack of resemblance to those of other fungi, it is determined that cytochrome p450 and transcription factors are the most prevalent [53]. In contrast to other fungi, *S. sclerotiorum* and *B. cinerea* lack terpene synthases, subtilases, zinc finger domains, and two glycosyl hydrolase families (GH2 and GH43) [48]. *S. sclerotiorum* has fewer genes encoding CAZymes than the majority of necrotrophic and hemibiotrophic fungi but considerably higher than the obligate biotrophic fungi *B. graminis* f. sp. *hordei* and *P. graminis* f. sp. *tritici* [115]. While entire CWDE profiles are comparable to those of saprotrophs, distinct CWDEs subsets/families may contribute to specific nectrotrophic habitats. Nevertheless, there is a correlation between gene conservation patterns and the emergence of a necrotrophic lifestyle. The expansion of gene families in *S. sclerotiorum*, *B. cinerea*, and two remotely related necrotrophic species, *P. teres* and *P. nodorum*, was investigated to identify traits that are shared among necrotrophic pathogens [53]. This analysis identified only heterokaryon incompatibility proteins.

Andrew and colleagues [128] investigated the possibility that members of the Sclerotiniaceae family have a group of genes related to necrotrophy in common. The phylogeny of 24 species reconstructed from the concatenated sequences of three housekeeping genes, *cal*, *hsp60*, and *g3ph*, agreed with the phylogenies derived from other loci [53]. The tree contained four major lineages. Both generalist and specialist host pathogens were found in two necrotrophic lineages: *Sclerotinia*/*Dumontinia*/*Sclerotium cepivorum* and *Botryotinia*/*Botrytis*. The third group comprises species that are either host specialists or facultative biotrophs (*Myriosclerotinia*). The necrotrophic, biotrophic, and host specialists, such as *Monilinia*, and the obligatorily biotrophic and host specialist *Ciborinia whetzelii* are members of the fourth lineage [128]. According to the conventional interpretation of this phylogeny, the progenitor of all members of the family Sclerotiniaceae was necrotrophyte, while biotrophy is considered a more recent, achieved state resulting from one or two modifications away from necrotrophy.

## 10. Conclusions

Over the past decade, uncovering the genomic features of *S. sclerotiorum* has facilitated comprehension of the intricacies of mechanisms underlying *S. sclerotiorum* growth, development, and virulence. Genetic evidence of the importance of OA, CWDEs, and effector proteins in various phases of pathogenesis has been unfolded. Comparative analyses demonstrate that necrotrophic infections do not encode much more quantity of degradative enzymes than other infection types, except for cutin degradation. Efforts are in progress to identify and characterize new genes involved in pathogen growth, development, pathogenesis, and stress adaptation. Moreover, the population of *S. sclerotiorum* appears to be more diverse than previously thought. Understanding the underlying reason for this divergence may help to understand Sclerotinaceae strategies for narrow versus broad host-range specialization. Previous *Sclerotinia* genome analysis may not have revealed definitive answers to all the underlying mechanisms of *S. sclerotiorum* biology and genetics, but it has already laid the groundwork for future research. Metabolomics studies that define fungal compounds, their regulation, and their roles in plant infection could help understand the specificity of plant–host connections even more. Functional genomic studies, comparative pathobiology and genome research, and a full assessment of population dynamics and structure are required to achieve further progress. Such efforts using genomic and genetic resources are expected to shed light on the intricate nature of the necrotrophic pathogenicity of *S. sclerotiorum*.

## Figures and Tables

**Table 1:** List of potential genes regulating growth, development, and virulence of *Sclerotinia sclerotiorum*.

Gene Name	Annotation	Gene Functions	Reference
*Sspac1*	pH-responsive transcription factor-encoding gene	Radial growth, sclerotial development (having the melanized rind layer), and virulence	[25]
*Sscna1*	Catalytic subunit calcineurin-encoding gene	Virulence	[81]
*PP2B*	Type 2B phosphatase	Maturation of sclerotium	[81]
*Ssnox1*	NADPH oxidase	Sclerotial formation	[82]
*SsITL*	Integrin-like protein	Virulence, hyphal polarity, and sclerotial formation	[64]
*Sssod1*	Cu/Zn-superoxide dismutase	Hyphal growth, sclerotial formation, virulence, oxidative stress tolerance, host ROS repression, and fungicide resistance	[83]
*SsMADS*	MADS-box transcription factor	Aerial growth, sclerotial development, and virulence	[84]
*Scat1*	Type A catalase	Radial growth, larger and melanized sclerotia, and osmotic stress tolerance	[73]
*Shk1*	Histidine kinase	Radial growth, sclerotial formation, oxidative stresses, and glycerol accumulation	[85]
*SsCVNH*	Cysteine-rich, small secreted protein with a carbohydrate-binding module	Normal mycelial growth and sclerotial formation	[86]
*Ssoah1*	Oxaloacetate acetylhydrolase	Radial growth, compound appressorium formation, sclerotial development, and pathogenesis	[31]
*Ssodc1*	Oxalate decarboxylase	Vegetative hyphae, apothecia, early phases of compound appressorium formation, and during plant colonization	[87]
*Ssodc2*	Oxalate decarboxylase	Middle-to-late phases of compound appressorium formation	[87]
*Ss-Bi1*	BAX inhibitor-1	Full virulence	[71]
*Ss-sop1*	Microbial opsin homolog gene	Radial growth, sclerotial formation, tolerance to salt, and osmotic and cell wall stresses	[68]
*Ss-Xyl1*	Endo-β-1, 4-xylanase	Normal colony morphology, radial growth, apothecia producing fertile sclerotial formation, and pathogenicity	[59]
*Ss-SSVP1*	Small secreted virulence-related protein	Slightly necessary for radial growth	[67]
*rhs1*	Rearrangement hotspot repeat-containing protein	Hyphal growth, sclerotial development, and virulence	[89]
*SCD1*	Scytalone dehydratase	Vegetative growth and sclerotial development	[26]
*THR1*	Trihydroxynaphthalene reductase	Vegetative growth and sclerotial development	[26]
*Ss-ams2*	A protein predicted to contain GATA-box domain	Radial growth, appressorium formation, pathogenicity, and chromosome segregation	[91]
*Ss-sfh1*	GATA transcription factor with an SNF5 domain	Radial growth, ROS accumulation, and virulence	[92]
*Ss-Ste12*	*Ste12* transcription factor	Mycelial growth, sclerotial development, appressorium formation, and penetration-dependent pathogenicity.	[37]
*Ss-svf1*	Survival factor 1 homologue	Appressorium formation and virulence	[93]
*Ss-rhs1*	Rhs repeat-containing protein	Sclerotial development and hyphal infection	[94]
*Ss-Fkh1*	Forkhead transcription factor	Hyphal growth, sclerotial formation, and tolerance to oxidative and osmotic stresses	[88,96]
*Ss-AGM1*	N-acetylglucosamine-phosphate mutase	Chitin production, cell wall integrity of mycelia, infection cushions, sclerotial formation, and virulence	[95]

**Table 2:** Comparative highlight of genomes of various fungal pathogens.

Genomes	Phylum	Nutrition Mode	Genome Size (Mb)	GC (%)	Chromosome	Genes	Proteins	Genes/ Genome	Reference
*Sclerotinia sclerotiorum* 1980	Ascomycota	Necrotroph	38.9	41.55	16	11,368	11,130	292.24	[53]
*Scleromitrula shiraiana* SX-001	Ascomycota	Necrotroph	39.0	38.85	n/a	11,327	11,327	290.44	[115]
*Botrytis cinerea* B05.10	Ascomycota	Necrotroph	42.6	42.00	18	14,262	13,703	334.79	[116]
*Magnaporthe oryzae* 70-15 (MG8)	Ascomycota	Hemibiotroph	41.0	51.59	7	13,184	12,989	321.56	[117]
*Colletotrichum higginsianum* IMI 349063	Ascomycota	Hemibiotroph	50.7	54.41	11	14,650	14,650	288.95	[118]
*Colletotrichum graminicola* M1.001	Ascomycota	Hemibiotroph	51.6	49.09	n/a	12,387	12,020	240.06	[119]
*Valsa mali* 03-8	Ascomycota	Necrotroph	44.7	49.35	13	11,284	11,284	252.44	[120]
*Valsa pyri* SXYL134	Ascomycota	Necrotroph	35.7	51.70	n/a	10,855	10,855	304.06	[120]
*Blumeria graminis* f. sp. *hordei* DH14	Ascomycota	Obligate biotroph	118.7	45.40	n/a	7061	6495	59.49	[121]
*Neurospora crassa* OR74A	Ascomycota	Saprotroph	41.1	48.23	7	10,785	10,785	262.41	[122]
*Fusarium graminearum* PH-1	Ascomycota	Hemibiotroph	38.0	48.15	4	14,145	14,143	372.24	[123]
*Aspergillus nidulans* FGSC_A4	Ascomycota	Saprotroph	29.8	50.37	8	10,597	10,534	355.60	[124]
*Parastagonospora nodorum* SN15	Ascomycota	Necrotroph	37.2	50.37	n/a	12,773	12,391	343.36	[125]
*Pyrenophora teres* f. sp. *teres* 0-1	Ascomycota	Necrotroph	33.5	50.9	n/a	12,103	11,799	361.28	[126]
*Puccinia graminis* f. sp. *tritici* CRL 75-36-700-3	Basidiomycota	Obligate biotroph	88.7	43.80	n/a	16,304	15,979	183.81	[127]
*Melampsora larici-populina* 98AG31	Basidiomycota	Obligate biotroph	109.8	41.00	18	17,140	16,372	178.05	[128]

## Data Availability

All data generated or analyzed during this study are included in this published article.
